# Nonverbal communication skills: New-era education needs of younger generation medical students

**DOI:** 10.12669/pjms.38.6.6058

**Published:** 2022

**Authors:** Manabu Murakami, Shigeki Jin, Akiko Takeuchi, Kotaro Matoba

**Affiliations:** 1 Manabu Murakami, MD, PhD. Center for Medical Education and International Relations, Faculty of Medicine, Hokkaido University, Kita 15, Nishi 7, Kita-ku, Sapporo, Hokkaido 060-8638, Japan; 2 Shigeki Jin, PhD, Department of Forensic Medicine, Faculty of Medicine, Hokkaido University, Kita 15, Nishi 7, Kita-ku, Sapporo, Hokkaido 060-8638, Japan; 3 Akiko Takeuchi, DDS, PhD. Department of Forensic Medicine, Faculty of Medicine, Hokkaido University, Kita 15, Nishi 7, Kita-ku, Sapporo, Hokkaido 060-8638, Japan; 4 Kotaro Matoba, MD, PhD. Department of Forensic Medicine, Faculty of Medicine, Hokkaido University, Kita 15, Nishi 7, Kita-ku, Sapporo, Hokkaido 060-8638, Japan

We read an interesting article related to medical education by Aziz et al. in 2021, describing that optimal use of nonverbal communication positively affects students’ learning environment.^1^ As international relations officers, we encourage students to participate in exchange programs to help them become culturally competent physicians. However, shy Asian students often find communicating with foreigners in English difficult due to the language barrier. As Piza et al. suggested, nonverbal communication skills, including facial expressions, gestures, postures, and empathic attitudes are important in establishing optimal educational environments for students and help them overcome language barriers.^2^ Although our research (i.e., exploration of students’ language learning skill needs) differs completely from that of Aziz et al.^1^ (i.e. exploration of students’ perspectives of nonverbal communication), we felt the results could be transferable.

We interviewed 23 first-year Japanese medical students, asking, “What kind of skills would you like to acquire when learning medical English?” Students’ discussions were recorded, transcribed, codified, and organized into four categories: 1. Communicating with foreigners regarding illness, symptoms, and treatments; 2. Understanding audio-visual information in clinical settings; 3. Acquiring information by reading textbooks; and 4. Academic presentation or writing using advanced medical terminology (Table-I). Students prioritized acquiring communication skills to improve interactions with foreigners and emphasized nonverbal communication in categories 1 and 2.

**Table-I:** Summary of interview survey about students’ needs.

Categories	Abilities Under the Categories.	Students’ Comments
1. Communication with foreigners regarding illness, symptoms, and treatments	Perform role-play scenarios and active pair-work with a native speaker	*“If I conduct simulated clinical examinations of migraine headaches and watch how a doctor speaks and asks questions as a native speaker, I could become more familiar with medical English.”*
2. Understanding audio-visual information in clinical settings	Comprehend subtitled movies and television series that use medical English in clinical settings	*“I was watching a German-dubbed version of Frozen, and I thought it was fun [to see how German expressions are used in scenes]. It might be helpful to watch international medical television series and listen to conversations, the specialized terminology, and expressions used in clinical settings.”*
3. Acquiring information by reading textbooks	Read basic publications or textbooks in English and shift gradually to reading advanced contents.	* “I think it is beneficial to be exposed to terminology [that includes medical English] on a daily basis. Reading the specific English text once a week is a good idea.”*
4. Academic presentation or writing using advanced medical terminology	Make academic presentations or demonstrate writing skills, conveying advanced and specialized knowledge.	*“We should get used to the [medical English] terms, instead of just suddenly being exposed to advanced academic writing.”*

Although Aziz et al.’s^1^ study focused on BDS students in Pakistan, it may apply to medical/dental students in various countries. Our exploratory study about generation gaps in 2021 revealed that younger generation students could not understand unspoken, unverbalized assumptions of older generation *teachers*, and hoped for better and closer communication with seniors.^3^ Appropriate education involving nonverbal communication skills may alleviate students’ stress regarding their learning environment.

Despite difficulties owing to the COVID-19 pandemic, we expect to continue investigating and promoting future student exchanges between Asian countries, including Pakistan and Japan, once the pandemic is controlled.

## Authors’ Contributions:

**MM:** Research concept, Design of the study, Prior literature review, Data collection, Data analysis, Data interpretation, Preparing initial draft, Approving final draft, Guarantor of the manuscript.

**SJ:** Research concept, Design of the study, Prior literature review, Data collection, Data analysis, Data interpretation, Revising initial draft, Approving final draft.

**AT:** Research concept, Design of the study, Prior literature review, Data interpretation, Revising initial draft, Approving final draft.

**KM:** Research concept, Design of the study, Prior literature review, Data interpretation, Revising initial draft, Approving final draft, Corresponding author of the manuscript.

## References

[1] Aziz A, Farhan F, Hassan F, Qaiser A (2021). Words are just noise, let your actions speak:impact of nonverbal communication on undergraduate medical education. Pak J Med Sci.

[2] Piza F, Piza P, Schwartzstein RM (2019). The power of nonverbal communication in medical education. Med Teach.

[3] Murakami M, Matoba K, Hyodoh H, Takahashi M (2021). Generation gaps in medical education:an exploratory qualitative study. J Pak Med Assoc.

